# Breastfeeding and parents’ socioeconomic status buffer dental developmental stress in female infants

**DOI:** 10.1093/emph/eoaf011

**Published:** 2025-06-13

**Authors:** Emily Moes

**Affiliations:** Department of Physician Assistant Studies, University of St. Francis, Albuquerque, NM, USA; Department of Anthropology, University of New Mexico, Albuquerque, NM, USA

**Keywords:** odontometrics, fluctuating asymmetry, sex-specific, growth investment

## Abstract

**Background and objectives:**

Linking adult health to early life is limited by a lack of retrospective biomarkers of stress tied to narrow windows of early development. Teeth serve as ideal data sources to examine early life because their hard tissues endure from infancy through adulthood as permanent records of developmental stress. This study examines if dental fluctuating asymmetry (FA) in permanent molars, a measure of instability and plastic responses to stress, is associated with biocultural factors during development.

**Methodology:**

Data were sourced from dental casts and health history records of 303 child participants of the longitudinal Burlington Growth Study. Dental FA was calculated from the first and second permanent molar intercuspal distances. Biocultural factors of parental, gestational, and childhood characteristics were grouped into latent dimensions using factor analysis of mixed data, then analyzed against FA using logistic regression separated by sex.

**Results:**

Breastfeeding and high and low parental socioeconomic status were associated with lower FA in females. No relationships were found between biocultural factors and FA in males.

**Conclusion and implications:**

The sex-specific results are likely due to differences in the nutritional needs of males and females during the first several postnatal months. Furthermore, dimorphism in energetic investment strategies, where males favor body growth while females favor system development, may be responsible for differences in how periods of physiological stress affect biological systems. These results argue for sex-specific investigations of stress biomarkers to better link early life with adult health.

## INTRODUCTION

Early life experiences significantly impact the development of health outcomes across the life course. This is because fetal and infant development is especially sensitive to environmental, social, and biological factors that influence physiology. The Developmental Origins of Health and Disease hypothesis predicts that physiological stress that occurs during development will elicit plastic responses in an individual’s biology to facilitate short-term survival [1–3]. This response has a trade-off in which such an alteration sets a phenotypic trajectory that leads to a greater risk of developing disease(s) later in life. The first 1000 days following conception are recognized as a critical period of development [4–6], yet not all factors during this time play an equal role in setting phenotypic trajectories.

Exposure timing is important when studying stress because it determines which of the body’s systems are affected, and to what extent. Furthermore, because systems and tissues within each system face different periods in which they are maximally sensitive to disruption [7, 8], plastic responses to stress are unlikely to be the same across organs. Therefore, when studying poor health outcomes, an understanding of tissue- or system-level development and responses to stress is crucial to identify potential early life factors that can have lifelong consequences. Many poor health outcomes, such as cardiovascular disease, cancer, and mortality, are the result of highly plastic physiological systems that respond to a multitude of stresses during development [1, 9]. Unfortunately, most of these systems lack retrospective biomarkers of stress that can be tied to early development, limiting how research can link early life experiences during specific windows to adult health.

Teeth have long been used to investigate the history of early life due to their well-understood development, hard tissue preservation, lack of remodeling, and their developmental canalization [10]. Because teeth are relatively protected against external disruption, stresses that affect the dentition likely also influence other less canalized systems, making teeth an ideal data source for reconstructing a timeline of the influences of early life stress. Plastic responses in the dentition depend on the stage of development at the time that stress occurs. The precise ages at which disruptions occurred can be reconstructed based on the well-documented stages of dental development from fetal life through childhood.

Developing teeth are known to be impacted by childhood experiences such as illness, nutrition levels, and parental socioeconomic status (SES) which correlate with changes to enamel growth [11–13] and tooth eruption timing [14–16]. Because of the connection between mothers and children during gestation and infancy, maternal stresses may be passed onto children through epigenetic effects [17, 18]. For example, maternal psychosocial and physical health [19–21] and exposures to ambient environment during pregnancy based on seasonality and high temperatures [11, 19, 22] have been linked with wider neonatal lines (dental microstructures recording a disturbance in enamel formation around the time of birth), reduced enamel thickness, and increased cusp asymmetry in deciduous teeth. Maternal age at childbirth has been associated with both earlier and later ages of deciduous tooth eruption [23, 24].

Here, fluctuating asymmetry (FA) in permanent teeth is used as an indicator of developmental plasticity at the time of tooth formation. FA is a random deviation from perfect symmetry, considered to be a measure of developmental instability resulting from physiological disruption [25]. It is viewed as a plastic response to an individual’s inability to buffer against energetic disturbances [26–28]. FA results from a combination of environmental stimuli, genetic input, and random developmental (or ‘noisy’) variation [29, 30]. Theoretical models indicate that FA results from an interaction between the environment and the effectiveness of developmental canalization [29, 31]. The inherent conflict between buffering capabilities and canalization results in plastic responses in developing systems as a tradeoff to promote survival. For example, energetic investment in dental development is reduced, rather than shunting energy from other more important systems or functions during instances of stress, resulting in alterations to dental phenotypes. Because dental FA of permanent teeth arises during infancy and childhood and is retained in adulthood, it is useful as a retrospective biomarker of systemic stress that coincides with adult health outcomes.

Dental FA in the permanent dentition has been used in research on a variety of biocultural contexts with the goal of examining the effects of stress during development [32–35]. Results have been inconsistent between contexts and types of stressors [36]. For example, high FA has been linked to maternal alcohol consumption, obesity, and smoking during pregnancy [34, 37], but not to birthweight [38] nor environmental radiation exposure [35]. Such research demonstrates that dental FA responds to some, but not all, instances of gestational and early life stress. This may be because much of this work does not link stressors to a discrete window of development when teeth are more susceptible to disruption.

Inconsistencies in the literature may also be partly due to the traits examined for FA. Tooth crown dimensions and shape are known to be under varying degrees of genetic control within and between each tooth class [39–41]. This results in varying degrees of susceptibility to developmental disruption and capacity for plasticity. Phenotypic expressions of molar cusps are more impacted by environmental stimuli as compared to crown size [39, 42], the common metric used in dental FA research. Previous studies on dental FA may have relied on traits too conserved for plastic responses to systemic stress. An alternative set of measurements, molar intercuspal distances, could provide a more faithful indicator of stress tied to early development.

This study tests whether early biocultural factors are associated with dental FA in cusps of permanent molars. For dental FA to be considered a retrospective biomarker of stress, it must first be associated with specific factors during early life that are connected to later health outcomes, such as SES [43], ethnicity/race [44], maternal health [45], prenatal environment [46], and infant nutrition [47]. Since previous research has connected maternal demographic and gestational stress to changes in dental microstructures and tooth eruption [11, 19, 22–24], such stresses may also be recorded in tooth shape. Because molar cusp development occurs during gestation and early infancy, the primary hypothesis is that stresses on mothers and infants during this time will be associated with increased dental FA.

## Materials and Methods

Data were sourced from the child participants of the Burlington Growth Study (BGS), curated by the University of Toronto Faculty of Dentistry, in Ontario, Canada. The BGS was conducted between 1952 and 1971 and consists of longitudinal craniofacial data and health histories of approximately 90% of the child residents of Burlington, Ontario [48]. Participants were involved with the BGS beginning between ages 3–12 years, until adulthood. The Burlington population during this time had a slightly higher income than the national average and was predominantly of European descent [48].

For the current study, the sample consists of *n* = 303 individuals (149 females, 154 males) who have serial dental casts of both their deciduous and permanent dentition. Casts of deciduous teeth were created between the ages of 3–6 years; casts of permanent teeth were created throughout childhood and adolescence. All individuals were from singleton births, and no sibling pairs were included. Life history information was sourced from the associated participant health histories. Use of this sample has been approved by the Burlington Growth Centre and was not considered human subjects research by the University of New Mexico’s Institutional Review Board.

### Health histories

Retrospective health histories were reported by the mother upon each participant’s initial visit as part of inclusion in the Burlington Growth Study (at age 3–6 years), then updated as needed during subsequent visits. From these health histories, all data were sourced about parental, gestational, and early childhood variables to test as measures of stress.

Parental information included the mother’s age at the child’s birth, education level, father’s occupation, group membership, and mother’s pregnancy history. Education and occupation were considered proxies for SES. Occupation was originally recorded as job descriptions or business locations (e.g. a specific manufacturing company). For the current study, occupations were binned following Canadian government standards [49]. Race/ethnicity were not recorded in the original study, rather parents were asked about their ‘Nationality’ which reflected European countries that were sources of migration waves during this time [50]. For analysis, nationality responses were binned following ethnic categories in Burlington, ON and the surrounding area during the 1950s [51]. This variable is considered as group membership. Pregnancy history is included as a possible stressor since previous miscarriages have been linked with maternal psychological stress during the succeeding pregnancies [52], and birth order has been associated with neonatal health outcomes [53].

Gestational and childhood variables included: year of birth, season of birth, birthweight, gestational length, early infant diet, and childhood illness history. Season of birth was determined from the birth month. Gestational length was originally reported as ‘term’ or the number of weeks the child was born early/late. For this study, gestational length was binned into ‘Early’, ‘Term’, and ‘Late’. Based on the American College of Obstetricians and Gynecologists standard, full term pregnancies are 39-40 weeks, therefore ‘Term’ was considered to include all individuals listed as term or one week early. All others were binned into the ‘Early’ and ‘Late’ categories accordingly. Early infant diet was recorded as presence/absence of any breastfeeding, its duration (binned into months), and formula type. Commercially available infant formula was not widespread during this time; instead, mothers often prepared homemade mixtures consisting of milk and carbohydrates [54]. Due to the range of milk types (e.g. whole, skim, evaporated, nondairy) and carbohydrate types (ex. corn syrup, Dextri-maltose) listed on the records, formula type was categorized as ‘Manufactured’ or ‘Homemade’. Common childhood illnesses of the early 1950s were documented for all participants; if afflicted, age at occurrence (in years) was often, though not always, listed. These illnesses included chickenpox, measles, pneumonia, rubella, tonsilitis, and whooping cough. Other illnesses or medical conditions were noted if present. For this study, illness history was recorded as the presence of any illnesses before the ages of 1 year and 3 years, in which illnesses prior to 3 years included any illness prior to 1 year.

### Dental metrics

**Figure 1.**
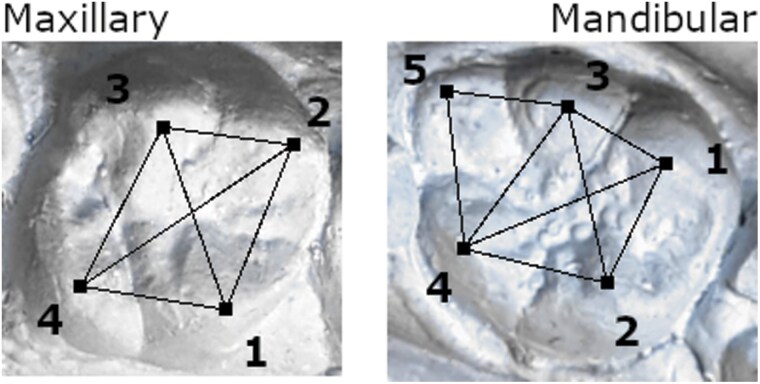
Intercuspal distances measured on the maxillary and mandibular molars. Cusp numbers correspond to names: Maxillary: 1—protocone, 2—paracone, 3—metacone, 4—hypocone; Mandibular: 1—protoconulid, 2—metaconid, 3—hypoconid, 4—entoconid, 5—hypoconulid. Although first and second molars were examined for asymmetry, only first molars are depicted here.

The intercuspal distances of the first and second permanent molars were measured using Hillson-Fitzgerald Mitutoyo digital calipers accurate to 0.01 mm (Fig. 1). Metric traits of third molars were not considered for this study due to their low presence in the sample, likely because they were unerupted as of the last age at casting or had been extracted. Because dentitions were cast at multiple ages, metrics were taken using the optimal cast for each trait, often shortly after the molar eruption. For this reason, age and attrition should not bias FA estimates.

### Preliminary analyses

Prior to calculating FA, several factors were evaluated that can bias FA estimates: intra-observer error, measurement error, irregular raw measurements, outliers, antisymmetry, directional asymmetry, size-shape dependency, and sexual dimorphism [55]. Intra-observer error was assessed by twice measuring 30 casts, with measurement events separated by at least 1 week. Paired *t*-tests were used to compare replicate measurements; metric traits with significant differences were excluded from further analyses. Using these replicate observations, traits were further excluded if mean measurement error (|measurement_2_—measurement_1_|) exceeded mean unsigned asymmetry (|R-L|) [55, 56].

Of the original 28 metric traits, six were omitted due to high intra-observer error or measurement error (Supplementary Table 1). An additional two traits were omitted due to the low presence of the hypoconulid of the mandibular second molar in the sample. The remaining 20 traits, listed in Table 1, were included in the final FA analyses as measures of developmental instability rather than the result of error. Before calculating the FA index, further preliminary analyses were conducted using the full sample of 303 individuals to ensure that measurements were appropriate [57]:

**Table 1:** Summative descriptions of a) parental, b) gestational, c) childhood, and d) FA characteristics separated by sex.

Variables	Males (*n* = 154)	Females (*n* = 149)	Variables	Males (*n* = 154)	Females (*n* = 149)
**a) PARENTAL CHARACTERISTICS**			**b) GESTATIONAL CHARACTERISTICS**		
Mother's age (yrs), mean (SD)	28.8 (4.76)	29.09 (4.52)	Year of Birth, *n*		
Mother's Period of Birth, *n*			1948	15	14
Prewar (1907–1914)	10	16	1949	20	37
WWI (1915–1919)	38	40	1950	56	49
Roaring 20s (1920–1929)	102	90	1951	62	46
Depression (1930–1934)	4	3	1952	1	2
Father's age (yrs), mean (SD)	31.7 (5.8)	31.9 (4.97)	1954	-	1
Mother's Education, *n*			Season of Birth, *n*		
< Grade 8	31	24	Winter	40	33
Some High School	53	38	Spring	43	46
Vocational School	18	25	Summer	32	40
Junior Matriculation	16	6	Fall	39	30
Senior Matriculation	11	24	Gestation Length, *n*		
Some University	13	17	Term	133	132
University	11	10	Early	9	8
Father's Education, *n*			Late	9	9
< Grade 8	35	25	Birthweight (kg), mean (SD)	3.40 (0.52)	3.33 (0.44)
Some High School	45	37			
Vocational School	14	19			
Junior Matriculation	12	12	**c) CHILDHOOD CHARACTERISTICS**		
Senior Matriculation	12	14	Breastfed, n	69	61
Some University	7	14	Breastfed Duration, *n*		
University	25	20	None—exclusively formula fed	85	88
Mother's Group Membership, n			<1 month	15	10
British or French	112	108	1–2 months	16	18
Irish	22	15	2–6 months	17	13
Other European	15	22	6 + months	21	20
Father's Group Membership, *n*			Formula Type, n		
British or French	112	97	None—exclusively breastfed	18	15
Irish	20	26	Homemade	108	116
Other European	18	20	Manufactured	24	14
Father's Occupation, *n*			Sick before 1 year, *n*	20	5
Business and Finance	19	13	Sick before 3 years, *n*	88	75
Natural and Applied Sciences	18	12			
Health	4	5			
Education, Law, and Social	6	6	**d) CHILD FA CHARACTERISTICS**		
Arts and Culture	4	6	FA-Dmdbl, mean (SD)	0.15 (1.02)	−0.13 (0.98)
Sales and Service	37	48	FA-gest, mean (SD)	0.04 (0.95)	−0.07 (1.03)
Trades, Transport, and Equipment	37	34	High molar intercuspal FA, *n*	25	20
Agriculture and Production	10	8			
Manufacturing and Utilities	18	15			
History of Prior Miscarriage, *n*	32	34			
Parity (before participant's birth), *n*					
Nulliparous	41	42			
primiparous	57	63			
multiparous	56	44

1. Assessment of outliers in the raw measurements by visual inspection of R-L scatterplots, followed by confirmation using Grubbs’ tests. Outliers were removed.
2. All traits were tested for patterned biases that could influence FA:
  a. Size-shape dependency and sexual dimorphism were tested using Spearman correlation between |R-L| and |(R + L)/2| in the pooled sample and when divided by sex, respectively.
  b. The presence of antisymmetry and directional asymmetry were evaluated by testing for significant deviations from normality.

After applying a sequential Bonferroni correction for multiple tests, there was no evidence of any of the above patterns that would influence FA.

### Calculating FA

FA was evaluated as aggregated measurements of the remaining 20 traits to increase its reliability as an indicator of physiological stress at the individual level rather than a measure of ‘developmental noise’ at the trait level [27, 28, 58]. First, each unsigned asymmetric trait was standardized using the full sample, then summed across traits. This value was then divided by the number of observed traits, since not all individuals had complete measurements. Individuals were excluded if they had fewer than ten traits to ensure a robust FA index without inflating values multiple standard deviations above the mean. As in previous work [22], this threshold was arbitrarily set to one half of the maximum number of traits, rounded up. The final sample size used for analysis was *n* = 297. The average number of measurements per individual was 17.8. Males had statistically more observable traits than females (*t*-test: mean for males 18.2, females 17.3; *t* = −3.91, df = 294.18, *P* = .00). Following Milne *et al*. [56], composite FA values were then z-standardized for ease of interpretation. The greater number of observable traits for males and females did not result in significant differences in z-standardized FA values (*t*-test: mean for males 0.05, females −0.08, *t* = −1.17, df = 292.01, *P* = .24). For analysis, FA was categorized as a dichotomous variable, following Yaussy [59], in which an individual had ‘high’ FA if their z-standardized FA index was greater than one, and received a value of 1. All other index scores were considered ‘low’ or ‘average’ and received a value of 0.

### Deciduous dental FA

Because gestational and early childhood variables are considered possible predictors of developmental instability in the permanent dentition, FA was calculated from deciduous teeth as a measure of prenatal and early infant developmental instability. Two indices were calculated. The first consisted of the mesiodistal and buccolingual diameters of all deciduous teeth (FA-Dmdbl). The second consisted of traits that develop only during gestation (FA-gest), which include mesiodistal diameters of incisors and molar intercuspal distances. See Moes *et al*. [22] for a complete description. Preliminary analyses to calculate both FA measures followed the same steps as above to assess error and measurement biases. The final index for FA-Dmdbl consisted of 15 traits, reflecting a developmental window from approximately 19 weeks in utero to 10 postnatal months based on the timing of enamel deposition for when crowns achieve their maximum diameters [60, 61]. For FA-gest, a total of 15 traits were included, reflecting a developmental window of 19–40 weeks in utero [60, 61].

### Data analysis

The relationship between 21 early life history variables and dental FA in permanent molar intercuspal distances was tested using R Statistical Software, version 4.1.2 [62], relationships were first explored using descriptive statistics, assessing the prevalence and distribution of each measure. Factor analysis of mixed data (FAMD) was used to explore underlying patterns in the early life history variables. FAMD is a dimension-reduction technique to quantify patterns in highly correlated datasets [63]. It is a combination of principal components analysis (PCA) and multiple correspondence analysis (MCA), allowing both continuous and categorical variables to be considered together. The analysis and resulting outcomes are interpreted similarly to PCA or MCA. For a robust sample size in dimension reduction, sexes were pooled for FAMD analysis, using the full *n* = 303.

Because FAMD cannot tolerate incomplete datasets, missing data were imputed using the ‘imputeFAMD’ function with five components to predict the missing values, as given by first using the ‘estim_ncpFAMD’ function [64]. Both functions are part of the ‘missMDA’ package [65]. Less than 5% of data were missing for any of the 21 variables (0–15 observations). Factor analysis on the complete dataset was performed using the ‘FactoMineR’ and factoextra” packages [66, 67].

The resulting dimensions were used in logistic regressions to test if latent characteristics of early life are associated with high dental FA. Because the results of FAMD analysis are used in the regressions, there is no multicollinearity between variables. Due to sample size reduction during FA analysis (see above), only *n* = 297 could be used in the logistic regressions, which were separated by sex (females = 146, males = 151). Model fit was examined via the residual deviance lack-of-fit statistic.

## RESULTS

### Participant characteristics

Descriptors of parental, gestational, and childhood characteristics are shown in Table 1. Children in the sample were born to parents with a mean age of approximately 30 years and were of British and French descent (Table 1a). Mothers and fathers had a wide range of educational experience, and fathers were employed across all occupation categories, although most were in sales and service, or in trades, transport, and equipment. Approximately 20% of the participants’ mothers had a history of prior miscarriage, and most had given birth to at least one previous child. Most children in the sample were born full term with normal birthweight, and with similar birth distribution across the seasons (Table 1b). Although formula-feeding infants during their first year was common practice in the 1950s [54, 68], approximately 42% of participants were breastfed for at least 1 week, with a duration of up to 6 months or longer (Table 1c). Few participants had recorded illnesses prior to their first birthday; approximately half had a recorded illness before reaching 3 years. Table 1d shows mean FA for the two indices with deciduous teeth (FA-gest, FA-Dmdbl) was near zero, although for both indices, mean FA is higher in males than females. This difference is significant for FA-Dmdbl (mean for males: 0.15, females: −0.13; *t*-test: *t* = −2.37, df = 300.99, *P* = .02). Approximately 15% of the sample (25 males, 20 females) had high FA in their permanent teeth, with no statistical difference between males and females (X^2^ = 0.47, df = 1, *P* = .49).

### FAMD analysis—interrelationships of life history variables

**Figure 2.**
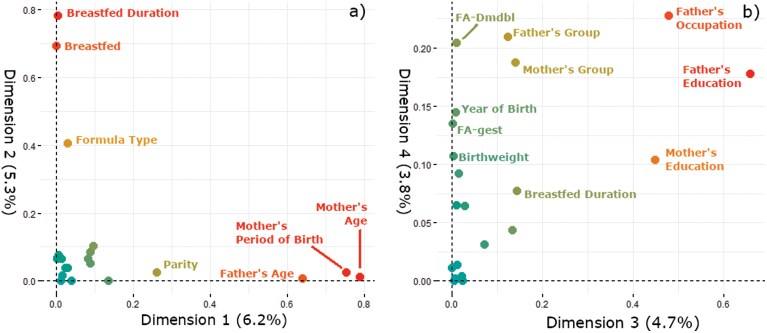
Individual variable contributions to a) dimensions 1 and 2 and b) dimensions 3 and 4. Colors correspond to heat mapping of relative contributions, where variables closer to the right and top of each graph have higher percent contributions.

The first four dimensions of the FAMD analysis explain 20% of the variance in the life history variables and are used for further analysis. A screeplot (Supplementary Fig. 1) was used to determine the number of dimensions to retain based on where the variances leveled off with each increase in dimension number. The first four dimensions were retained since there is little variance change in the fifth through tenth dimensions as compared to the fourth. The first dimension carries 6.2% of the variance; the fourth carries 3.8%. Figure 2 shows the variables that contribute most to each dimension. The combination of highest contributing variables to each dimension suggests that the following latent characteristics are described by each dimension: dimension 1—parents’ age at child’s birth (mother’s age, father’s age, mother’s time period of birth); dimension 2—breastfeeding history (breastfeeding presence, breastfeeding duration, formula type); dimension 3—parents’ SES (mother’s education, father’s education, father’s occupation); dimension 4—parental condition (father’s occupation, father’s education, mother’s nationality, father’s nationality, FA-Dmdbl, FA-gest, participant’s year of birth, birthweight, mother’s education).

**Figure 3.**
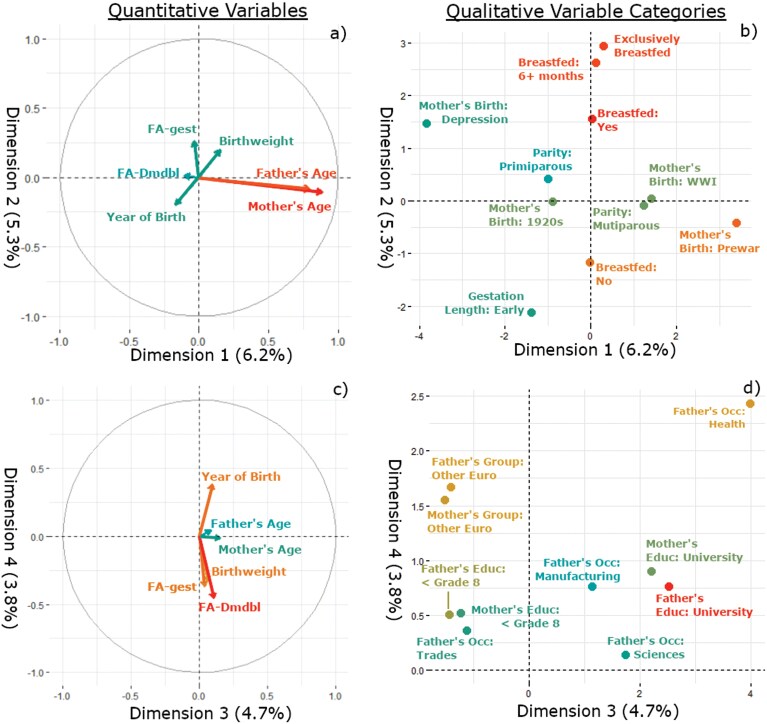
Highest contributing variables to dimensions 1–4 based on correlations for quantitative variables (left column) and categories for qualitative variables (right column). Panels a) and b) display variables for dimensions 1 and 2. Panels c) and d) display variables for dimensions 3 and 4. For correlation circles, the longer the vector arrow, the higher the correlation with the dimension(s). Relative contribution to each dimension also displayed as heat mapped colors.

Figure 3 shows the specific contributions of each salient variable for the quantitative (Fig. 3a and 3c) and qualitative variables (Fig. 3b and 3d). Dimension 1 is positively correlated with higher ages of parents, as shown by the correlation circle (Fig. 3a), and higher contributions of earlier time periods for mothers’ birth (Fig. 3b). Dimension 2 is highly correlated with the presence of breastfeeding, and for longer durations (Fig. 3b). Dimension 3 is strongly influenced by higher SES, as given by university-educated parents, and fathers with health- or science-based occupations (Fig. 3d). Low education and manual labor occupations are negatively associated with dimension 3. Dimension 4 is negatively correlated with measures of gestational and early life physiological stress (FA in deciduous teeth, birthweight), but positively associated with year of birth, parents with nationalities outside of the UK and France, parents with high and low education, as well as fathers with health-related occupations. Together, these variables are loosely interpreted to represent high and low parental SES. Parents of children in this sample with nationalities from ‘other European countries’ are more likely to have less education and have manual labor-based occupations (Supplementary Tables 2 and 3). They are also more likely to have children with low FA in their deciduous teeth (ANOVA: mean for UK and France: 0.1; Ireland: 0.00; other European countries: −0.39; *F* = 3.93; *P* = .02). Dimension 4 suggests that children’s early life stresses are associated with parents’ experiences at both ends of the socioeconomic spectrum, but not the middle.

### Regression—Predictors of high FA

Table 2 displays the results of the logistic regression for males and females, testing the association between the FAMD dimensions and the likelihood of having high intercuspal molar FA. None of the dimensions are significant predictors of high FA in males. In females, both dimensions 2 (breastfeeding history) and 4 (parental condition) are significant predictors (*P* = .02 and *P* = .005, respectively), when controlling for dimensions 1 (parents’ ages) and 3 (parents’ SES). The model residual deviance for lack-of-fit *P*-value is .99, indicating the model fits these data reasonably well. The beta coefficients for both dimensions 2 and 4 are negative, indicating that increases in breastfeeding experience and parental low and high SES significantly decrease the likelihood of females having high FA in their molar intercuspal distances.

**Table 2.** Logistic regression results separated by sex, displaying the regression coefficients and *P*-values for each variable. Note: Bolded values indicate *P* < .05.

Model Variables	High FA ~ dim 1 + dim 2 + dim 3 + dim 4			
	Males		Females	
	coeff. (SE)	*P*	coeff. (SE)	*P*
Intercept	−1.71 (0.24)	**<.001**	−2.38 (0.37)	**<.001**
Dimension 1	−0.21 (0.14)	.13	0.15 (0.16)	.34
Dimension 2	0.02 (0.14)	.85	−0.51 (0.22)	**.02**
Dimension 3	0.09 (0.15)	.51	0.03 (0.21)	.87
Dimension 4	−0.16 (0.18)	.36	−0.63 (0.22)	**.005**
lack of fit *P*	.8		.99	

## Discussion

The goal of this study was to determine if biocultural factors are associated with dental FA in molar cusps of the permanent dentition, a measure of physiological disruption recorded early in development. When considering gestational, parental, and early childhood influences, results indicate that dimensions capturing breastfeeding experience and parents’ status at the high and low ends of the socioeconomic spectrum significantly decrease the likelihood of having high FA, but only in females.

Breastfeeding and formula feeding are known to have pronounced differences on infant development [68–70]. For example, formula-fed infants gain weight and length faster than do their breastfed counterparts, even in the first week of life [71, 72]. Despite these initial benefits, breastfeeding may have later protective effects against chronic illnesses like obesity, hypertension, dyslipidemia, and type II diabetes [73]. The infant formulas fed to the participants in this study were either homemade mixtures of evaporated milk, water, and a carbohydrate (corn syrup), or commercially manufactured powders or concentrated liquids. Although calorie-dense, such formulas during the 1950s were associated with infant nutritional deficiencies and imbalances, such as low iron content, high protein, low essential fatty acids, and low vitamin C [54].

Despite these imbalances, the results suggest that breastfeeding only had a protective effect on the dental development of females, rather than both sexes. The sex-based differences in response to early environment support findings that there may be sex-specific requirements during the earliest phase of life for optimal growth and development [74–76]. Likely, this is due to differences in nutritional needs between male and female infants for optimal growth and development during the first few months of postnatal life, as indicated by composition differences in breastmilk [74, 75]. Case studies in Singapore and Massachusetts have found greater caloric density in the breastmilk of mothers with male infants as compared to females [77, 78]. Similarly, when examining macronutrients, a study in Poland found a higher content of carbohydrates in breastmilk for males than for females [79]. This pattern may be dependent on SES; poor mothers in a study in Northern Kenya produced breastmilk with higher fat concentration for their daughters than for their sons, but this relationship was reversed for economically sufficient mothers [80]. Given the context of the participants in the Burlington Growth Study where differences in SES were not as pronounced [48], it is unlikely that SES interacted in a similar way to influence sex-based breastmilk composition. Instead, it may be that the energy density in breastmilk for males was comparable to that in formula milk, thereby reducing physiological stresses that could have impacted their dental development. Conversely, the nutritional needs for formula-fed females may not have been adequately met, requiring other nutrients besides caloric density, resulting in physiological disruption or imbalance that also affected their dental development.

Breastfeeding, regardless of its duration, may have provided an advantage in the developmental stability of females’ growing dentitions. Molar intercuspal FA was likely affected due to (i) the traits’ heightened sensitivity to disruption [39, 42] and (ii) the overlap with key periods of first and second molar development. Although the initial locations of first molar cusps are established during the fourth and fifth fetal months [81], their distances increase until the slopes between cusps are calcified by dentin deposition [39, 82], which occurs throughout the first postnatal year. Simultaneously, the tooth buds of second molars are developing during this time [83], therefore nutritional stresses from formula feeding may have influenced the scaffolding stages of crown and cusp development.

Of the gestational, parental, and early childhood variables that comprised the FAMD analysis, the dimension capturing parents’ marginal SES was the only other significant predictor of intercuspal FA. Parents with low or high socioeconomic status had daughters that were significantly less likely to have high FA. Low SES in this dimension was represented by the following variables: parental education less than grade eight, nationalities from European countries outside of the United Kingdom and France, and fathers with manufacturing and trades occupations (Fig. 3d). High SES was represented by university-educated parents, and fathers who worked in health- or science-related fields. Burlington, Ontario and the surrounding area during the 1940s and early 1950s saw a sizeable population increase driven by European immigrants attracted to the manufacturing industry [84]. Nationality shaped the opportunity structure for residents of the area, in which working-class neighborhoods came to be defined by different income groups and migration origins outside the United Kingdom and France [84]. Industrial workers, especially those of eastern and southern European descent, were mostly living in the highly polluted neighborhoods near the steel mills while wealthier residents lived in more desirable neighborhoods far from the industrial areas [84–86]. As a result, individuals primarily from eastern and southern Europe, who were likely fleeing during and after WWII, were subject to social ‘othering’, further influencing the social and economic opportunities available to them [51, 85].

Based on these trends, the parents in the current study with ‘Other European’ nationalities were likely recent or first-generation immigrants with low SES, yet their daughters had a significantly lower risk of developing high FA. This is consistent with the ‘healthy migrant’ effect [87, 88]. Despite the challenges of migrating and living in a new country, immigrants from socioeconomically poor places are often observed to have comparatively better health than the local residents [89, 90]. This benefit has been shown to pass to the next generation when considering birth outcomes [87, 88], although the benefit is fleeting, often only lasting a single generation [89, 91]. It may be that ‘Other European’ parents with low education attainment and manual labor-based occupations were first-generation immigrants benefitting from the decreased social, environmental, and/or economic stresses that caused them to migrate. Furthermore, they may not have yet embodied the varied stresses of industrialized Canada. For example, first-generation immigrants often continue their familiar cultural and dietary practices from their home countries, acting as buffers against local influences including against low economic status [92]. As a result, mothers in this study may have passed on the buffering benefit to their daughters during gestation and early infancy, resulting in lower FA. At the same time, parents with higher SES were likely buffering their daughters from developmental disruption via mechanisms tied to their wealthier backgrounds in the local environment, consistent with high SES benefits on gestational and infant health [93].

The lack of significant effects on FA in males may be because developmental disruption caused by the social, cultural, and environmental impacts in this context were not severe enough to impact their dentition. Instead, male physiology during gestation and early infancy prioritize high investment in growth [94], so disruption during this time could have impacted other systems such as those associated with weight gain or body growth. This is consistent with previous research finding changes in skeletal development in males but not females in response to fetal stress measured by birthweight [95]. By comparison, females, whose energetic investment focuses more on developmental reserve and immune responses, face comparatively restricted growth investment [96, 97]. Therefore, during periods of physiological stress, females may experience disruptions to other developing systems (like dentition) rather than shunting energy away from areas already restricted [98].

Future research should consider sex-specific biomarkers of stress during infancy, such as changing velocities in weight gain or length among males, to track physiological disruptions in response to early environments. Investigating sex-specific responses to stress during gestation and early infancy may provide critical insight into the evolution of plasticity and phenotypic variation. In turn, this will allow us to further explore the mechanisms responsible for adverse adult health outcomes.

### Limitations

This study relies on retrospective health questionnaires given to the parents of the growth study participants shortly after they became involved, when participants were 3–6 years of age. For many parents, there could have been recall bias in the details included in their responses, especially regarding questions of gestational experiences and early infant health. Additionally, because of the open-ended nature of the questions, responses were not standardized, nor did they consistently include the same information across participants, which made data analyses reliant on binned categorical variables to maximize sample sizes for each environmental factor under consideration. In this way, key sources of variation in stressors may have been homogenized.

## Conclusion

The goal of this study was to test if dental FA in permanent teeth is a marker of early life stress tied to biocultural factors. The results suggest that this relationship exists in females but not in males, given the variables considered. The sex-biased results are consistent with previous work from this project focused on FA in deciduous teeth which found that FA in females is tied to ambient environmental temperature during gestation [22]. Together, these studies support research showing that fetal and early infant susceptibility to environmental stressors differs between males and females, likely based on sex-specific patterns in growth investment strategies as well as different windows of heightened sensitivity during development [94, 99]. It may be that when some developing biological systems are under relatively increased investment (e.g. body growth vs. dental development), energetic resources will be shunted from these rather than reducing investment in the systems already restricted. Additional work is needed to understand how investment strategies are prioritized as the body moves through sensitive windows of development, especially as they interact with the high energetic demands of the brain in early life. Such work would advance our understanding of sex-specific plastic responses to disruption and how stress is embodied during development. Although dental FA may be an indicator of early life stress in females, future research should explore other indicators in males that may serve as retrospective markers during adulthood of early life stress, such as those that may affect skeletal development.

## Supplementary Material

eoaf011_suppl_Supplementary_Tables_S1-S3_Figure_S1

## Data Availability

The data underlying this article are available in the article and in its online supplementary material.
