# Supplementary material for: Breastfeeding and parents’ socioeconomic status buffer dental developmental stress in female infants
Source: Evol Med Public Health. 2025 Jun 13;13(1):140–53. doi: 10.1093/emph/eoaf011 (PMC12238712; doi:10.1093/emph/eoaf011)
Supplement: eoaf011_suppl_Supplementary_Tables_S1-S3_Figure_S1 [file eoaf011_suppl_supplementary_tables_s1-s3_figure_s1.docx]

Supplementary Tables and Figures

Supplementary Table 1. Maxillary and mandibular molar intercuspal traits retained and omitted for final FA calculations. Numbers in parentheses represent cusp numbers shown in Figure 1. a – omitted due to high intra-observer error; b – omitted due to high measurement error; c – omitted due to lack of observations

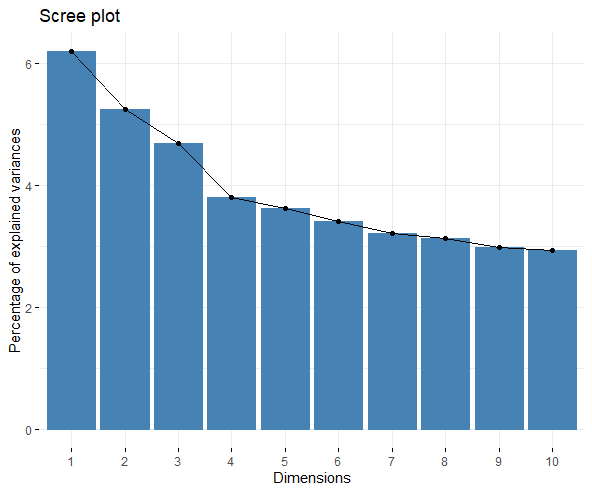


Supplementary Figure 1: Screeplot from FAMD results. The first four dimensions were retained for further analysis.

Supplementary Table 2: Frequency table displaying father’s nationality by education level. Pearson’s Chi-squared test: X^2^ = 21.88, df = 12, p = 0.03

| **Frequency Table: Father's Nationality vs Education** | | | | | | | |
| --- | --- | --- | --- | --- | --- | --- | --- |
|  | < Grade 8 | Some HS | Vocational | Junior Matric. | Senior Matric. | Some University | University |
| UK - France | 38 | 59 | 19 | 16 | 19 | 18 | 34 |
| Ireland | 7 | 11 | 10 | 6 | 1 | 2 | 6 |
| Other Euro | 13 | 10 | 3 | 2 | 5 | 0 | 3 |

Supplementary Table 3: Frequency table displaying father’s nationality by occupation classification. Pearson’s Chi-squared test: X^2^ = 11.28, df = 16, p = 0.79

| **Frequency Table: Father's Nationality vs Occupation** | | | | | | | | | |
| --- | --- | --- | --- | --- | --- | --- | --- | --- | --- |
|  | Business | Sciences | Health | Education | Arts | Sales | Trades | Agriculture | Manufacturing |
| UK - France | 19 | 22 | 7 | 8 | 8 | 64 | 43 | 13 | 24 |
| Ireland | 7 | 3 | 2 | 2 | 2 | 12 | 12 | 1 | 4 |
| Other Euro | 5 | 2 | 0 | 2 | 0 | 9 | 13 | 3 | 4 |
